# High‐Performance Se–S Composite Cathode Rich in Defects for Wide‐Temperature Solid‐State Lithium Batteries

**DOI:** 10.1002/smsc.202300134

**Published:** 2023-11-20

**Authors:** Xiaomeng Shi, Zhichao Zeng, Yongqing Wen, Hongtu Zhang, Yabin Zhang, Yaping Du

**Affiliations:** ^1^ Tianjin Key Lab for Rare Earth Materials and Applications Center for Rare Earth and Inorganic Functional Materials Smart Sensing Interdisciplinary Science Center School of Materials Science and Engineering National Institute for Advanced Materials Nankai University Tianjin 300350 China; ^2^ Baotou Research Institute of Rare Earths Rare Earth Advanced Materials Technology Innovation Center Baotou 014010 China; ^3^ State Key Laboratory of Featured Metal Materials and Life-cycle Safety for Composite Structures MOE Key Laboratory of New Processing Technology for Nonferrous Metals and Materials, and School of Resources Environment and Materials Guangxi University Nanning 530004 China; ^4^ Haihe Laboratory of Sustainable Chemical Transformations Tianjin 300350 China

**Keywords:** halide solid electrolytes, lithium batteries, rare earths, Se_*x*_S_1–*x*_ composites, solid-state batteries

## Abstract

All‐solid‐state lithium batteries (ASSLBs) are a research hotspot for their superior safety. The solid electrolytes (SEs) are key components in ASSLBs, and the emerging rare‐earth halide SEs (RE‐HSEs) are valued for their comprehensive performances of good ionic conductivity, electrochemical stability, and deformability. In addition, cathode materials can influence the properties of ASSLBs, and sulfur (S) attracts much attention due to the lower toxicity and much higher energy density compared with commercial oxide cathodes. However, the S possesses poor electronic conductivity, which can be improved by the introduction of selenium (Se) with much higher electronic conductivity. In this work, a series of Se_*x*_S_1–*x*_ composites is synthesized by a melting method. Due to the introduction of Se and the enriched defects from the melting process, the electronic and ionic conductivities of Se_*x*_S_1–*x*_ are improved. After application in ASSLBs based on RE‐HSE Li_3_YBr_6_, the Se_*x*_S_1–*x*_ materials exhibit good performances with low polarizations, good cycling stabilities, and excellent rate properties at room temperature. Moreover, the assembled solid batteries can realize stable cycling performance (100 cycles) at low temperature (−30 °C) and a normal discharge–charge process at high temperature (120 °C).

## Graphical abstract

A series of selenium and sulfur composites (Se_*x*_S_1–*x*_) with improved electronic/ionic conductivities is prepared. The all‐solid‐state lithium battery with the Se_*x*_S_1–*x*_ cathode exhibits good cycling stability (250 cycles), large reversible capacity, excellent rate performance (0.1–10C), and wide operating temperature range (−30–120 °C).

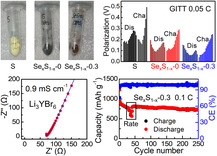

## 1 Introduction

On account of the high safety and energy density, all‐solid‐state lithium batteries (ASSLBs) have been a research hotspot.^[^ ^1^, ^2^, ^3^, ^4^, ^5^ ^]^ The safety can be guaranteed by stable solid electrolytes (SEs)^[^ ^6^, ^7^, ^8^, ^9^, ^10^, ^11^, ^12^ ^]^ and the energy density is influenced by the type of cathode.^[^ ^13^ ^]^ Among the different cathode materials, sulfur (S) exhibits low toxicity, high abundance, and especially large theoretical capacity (1675 mAh g^−1^).^[^ ^14^, ^15^, ^16^ ^]^ In addition, the shuttling effect of lithium polysulfide intermediates can be avoided in ASSLBs.^[^ ^17^ ^]^ Thus, solid lithium–sulfur (Li–S) batteries have been valued by researchers.^[^ ^18^, ^19^, ^20^, ^21^, ^22^ ^]^ For the researches on solid Li–S batteries, sulfide SEs are usually adopted due to the high ionic conductivity and good compatibility with S/Li_2_S cathodes. However, the narrow electrochemical windows of sulfide SEs^[^ ^23^, ^24^, ^25^, ^26^, ^27^ ^]^ have hindered the applications of solid Li–S batteries. In recent years, rare‐earth (RE) halide SEs (RE‐HSEs) have attracted much attention for the comprehensive advantages of ionic conductivity, deformability, and electrochemical stability.^[^ ^28^, ^29^, ^30^, ^31^, ^32^, ^33^, ^34^, ^35^, ^36^, ^37^, ^38^ ^]^ Moreover, the RE‐HSEs possess rich adjustability and high structural stability originating from the abundant and large ion radii, low electrode potentials, and small electronegativities of RE elements.^[^ ^39^, ^40^, ^41^, ^42^, ^43^ ^]^ In addition, these SEs have good compatibility with chalcogenide cathode materials.^[^ ^44^, ^45^, ^46^, ^47^ ^]^ Thus, the RE‐HSEs can achieve promising applications in Li–S batteries.

Up to now, the researches on RE‐HSEs applied in Li–S batteries were limited and they exhibited large discharge–charge polarizations and poor rate performances at a high temperature of 60 °C.^[^ ^44^ ^]^ As a member of the same family of S, selenium (Se) exhibits superior electronic conductivity (Se: 1 × 10^−5^ S cm^−1^; S: 5 × 10^−30^ S cm^−1^) and similar chemical properties to S.^[^ ^48^ ^]^ In addition, some works have selected Se material^[^ ^46^, ^48^, ^49^ ^]^ as a cathode to realize high efficiency and good cycling and excellent rate performances at room temperature (RT); however, the theoretical capacity was decreased obviously (Se, 675 mAh g^−1^). Thus, Se can be introduced into the S material to form Se_*x*_S_1–*x*_ composites, which can exhibit high electronic/ionic conductivities and maintain large battery capacities.^[^ ^50^ ^]^ In addition, the ASSLBs with RE‐HSEs possess the promising applications over a wide‐temperature range for the good ionic conductivity and thermal stability of RE‐HSEs.

In this work, a series of Se_*x*_S_1–*x*_ (0 ≤ *x* ≤ 0.9) composites were prepared by melting method (380 °C). Benefiting from the introduction of Se and the melting process, the electronic/ionic conductivities of synthesized Se_*x*_S_1–*x*_ materials can be improved. Therefore, the Se_*x*_S_1–*x*_ exhibited low discharge–charge polarizations, and good cycling and rate performances with high capacities in ASSLBs based on Li_3_YBr_6_ (LYB) electrolyte at RT. In addition, the assembled solid batteries can be operated over a wide‐temperature range (−30–120 °C) for the high stability and ionic conductivity of LYB and good electronic/ionic conductivity of the Se_*x*_S_1–*x*_ cathode. Therefore, this optimization method for the S cathode can promote the practical application of ASSLBs with RE‐HSEs.

## 2 Results and Discussions

**Figure 1:**
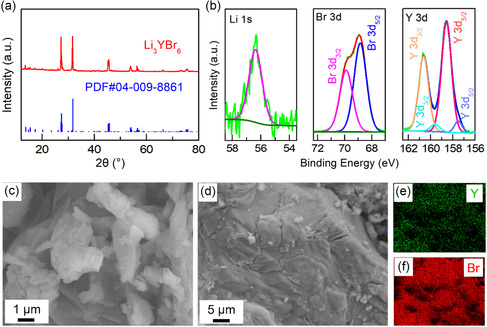
Properties and characteristics of Li_3_YBr_6_ (LYB). a) X‐ray diffraction (XRD) pattern of synthesized LYB sample. b) X‐ray photoelectron spectroscopy (XPS) spectra of LYB. c,d) Scanning electron microscopy (SEM) images of LYB and e,f) the corresponding energy‐dispersive spectroscopy (EDS) mapping of Y and Br.

In this work, the reported halide material LYB was selected as the SE. The LYB sample was obtained by a simple vacuum evaporation method reported in our previous works.^[^ ^45^ ^]^ From the X‐ray diffraction (XRD) pattern (**Figure** **1**a), the obtained diffraction peaks of the synthesized LYB sample are well indexed with the PDF card (PDF#04‐009‐8861), indicating that LYB has been synthesized successfully. According to the X‐ray photoelectron spectroscopy (XPS) results, the valence state of Li^+^, Y^3+^, and inorganic Br^−^ in LYB can be confirmed (Figure 1b). In addition, the LYB sample possesses micro‐sized particles with uniform distribution and appropriate proportion of Y/Br elements from the scanning electron microscopy (SEM) and energy‐dispersive spectroscopy (EDS) results (Figure 1c–f, Figure S1, Supporting Information). To study the electrochemical properties of the synthesized LYB sample, electrochemical tests were performed on assembled LYB based cells. From the electrochemical impedance spectroscopy (EIS) test, the Nyquist plot of indium (In)/LYB/In cell can be obtained (Figure S2, Supporting Information), and the calculated ionic conductivity of LYB is 0.9 mS cm^−1^. In addition, the carrier of the LYB sample can be confirmed as lithium ion according to the instantaneous current tests performed on Li/Li_7_P_3_S_11_ (LPS)/LYB/LPS/Li and SG/LYB/SG (SG, stainless‐steel gasket) cells (Figure S3, Supporting Information). Thus, the synthesized LYB sample is suitable for the application of solid‐state battery. Furthermore, the compatibility between LYB and anode was evaluated by discharge–charge tests on Li/LYB/Li and Li–In/LYB/Li–In cells (Figure S4, Supporting Information). The Li metal is unstable with the LYB sample, which induces large polarization in the Li/LYB/Li cell. Fortunately, the LYB has good stability with the Li–In alloy for the stable and small voltage polarization (≈11 mV). Therefore, the Li–In alloy anode was adopted in the LYB‐based ASSLB.

**Figure 2:**
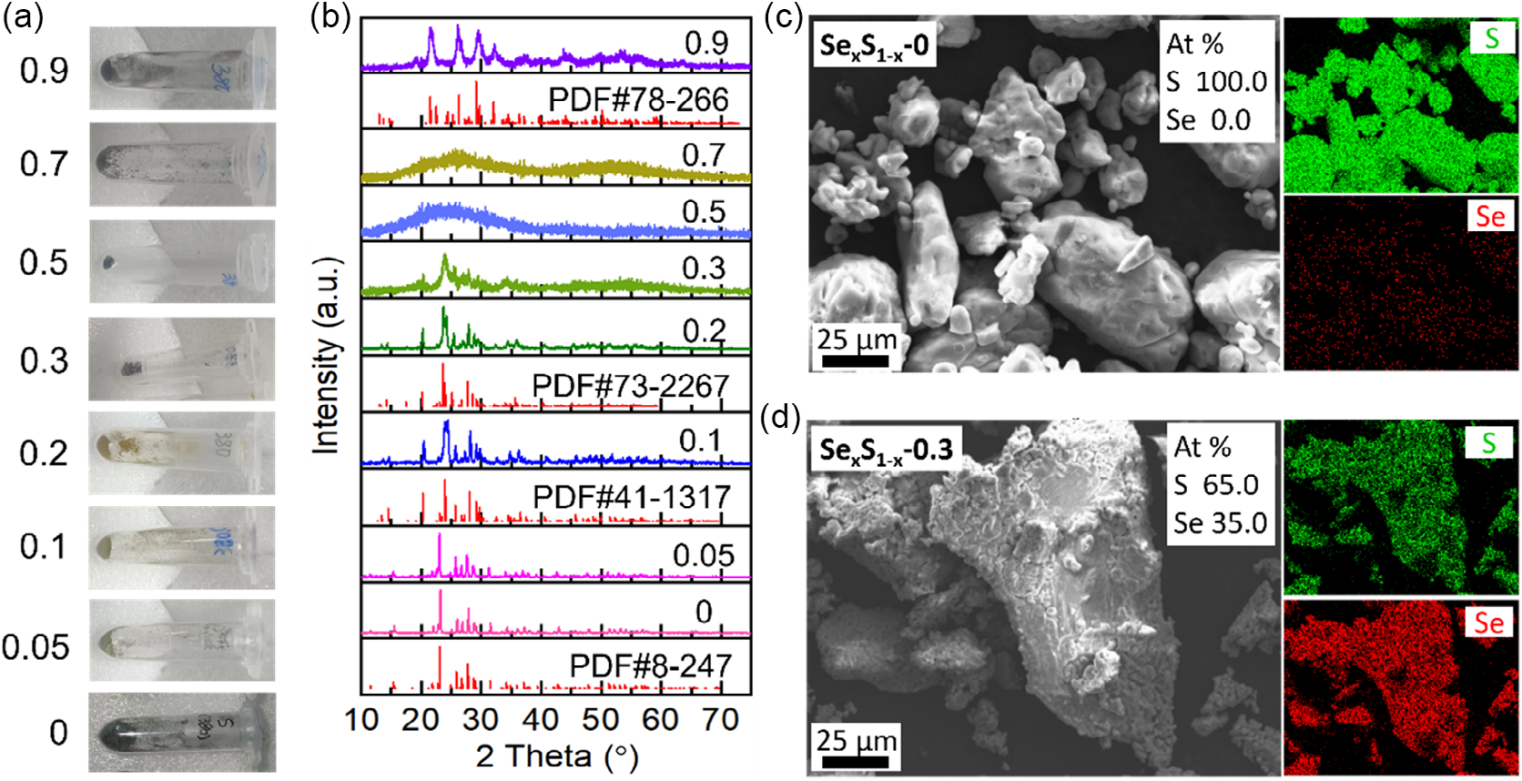
The structure and morphology of Se_*x*_S_1–*x*_. a) Photographs and b) XRD patterns of prepared Se_*x*_S_1–*x*_ (0 ≤ *x* ≤ 0.9) materials (S, PDF#8‐247; S_27.64_Se_4.36_, PDF#41‐1317; Se_2.57_S_5.43_, PDF#73‐2267; Se_5.1_S_1.9_, PDF#78‐266). c,d) SEM and EDS results of prepared Se_*x*_S_1–*x*_‐0 (c) and Se_*x*_S_1–*x*_‐0.3 (d) materials.

The large theoretical capacity and suitable operating voltage of the S cathode are beneficial to the performances of LYB‐based solid Li–S batteries. However, the poor electronic conductivity of S hinders the practical application of solid Li–S batteries. To overcome this shortcoming of S, Se with high electronic conductivity was introduced into the S material by the melting method (380 °C), and a series of Se–S composite materials, Se_*x*_S_1–*x*_ (0 ≤ *x* ≤ 0.9), were prepared. According to the photos of the S‐based materials (**Figure** **2**a, Figure S5–S6, Supporting Information), the yellow commercial S powder turned black after the melting process. Along with the increase in the Se ratio, the color of Se_*x*_S_1–*x*_ gradually turned to brick red with increased viscidity. When *x* = 0.3, the Se_*x*_S_1–*x*_ sample was black and viscoelastic, which became hard after standing process and could be ground to brick red powder. When *x* = 0.5, the black viscoelastic sample with increased hardness can maintain its viscidity after standing for a long time. When *x* > 0.7, the obtained Se_*x*_S_1–*x*_ sample lost the viscidity and could be ground to black powder. After that, the structure of the Se_*x*_S_1–*x*_ sample was analyzed by XRD (Figure 2b, Figure S7–S8, Supporting Information). The collected XRD patterns of S and Se_*x*_S_1–*x*_ (*x* = 0) (named as Se_*x*_S_1–*x*_‐0) were similar and could be indexed to the PDF card (S, PDF#8‐247) (Figure 1e). Thus, the melting process almost has no effect on the crystal phase of S sample. When *x* = 0.05, the XRD pattern of Se_*x*_S_1–*x*_ sample was similar with S and Se_*x*_S_1–*x*_‐0 with decreased intensity. Along with the continued increase in the *x* value, the structure of Se_*x*_S_1–*x*_ sample changed from the orthorhombic phase to the monoclinic phase (*x* = 0.1, PDF#41‐1317 [S_27.64_Se_4.36_]; *x* = 0.2 and 0.3, PDF#73‐2267 [Se_2.57_S_5.43_]) with further decreased crystallinity. When *x* = 0.3, the XRD signal almost disappeared, and could be recovered partially after standing process (Figure S2, Supporting Information). When *x* = 0.5 and 0.7, the XRD signal of the Se_*x*_S_1–*x*_ sample disappeared, indicating that the sample possessed an amorphous structure. When *x* = 0.9, the XRD peak of the material appeared and the structure of the Se_*x*_S_1–*x*_ sample turned to the orthorhombic phase (Se_5.1_S_1.9_, PDF#78‐266). In addition, the morphology and constituent of Se_*x*_S_1–*x*_ samples were characterized (Figure 1c,d, Figure S9, Supporting Information). From the SEM/EDS results, the Se_*x*_S_1–*x*_ materials possess abnormal micro‐sized particles with uniformly distributed elements of Se and S. Moreover, the molar ratios of Se/S were close to the theoretical value, and a small amount of S may be lost during the synthetic processes.

**Figure 3:**
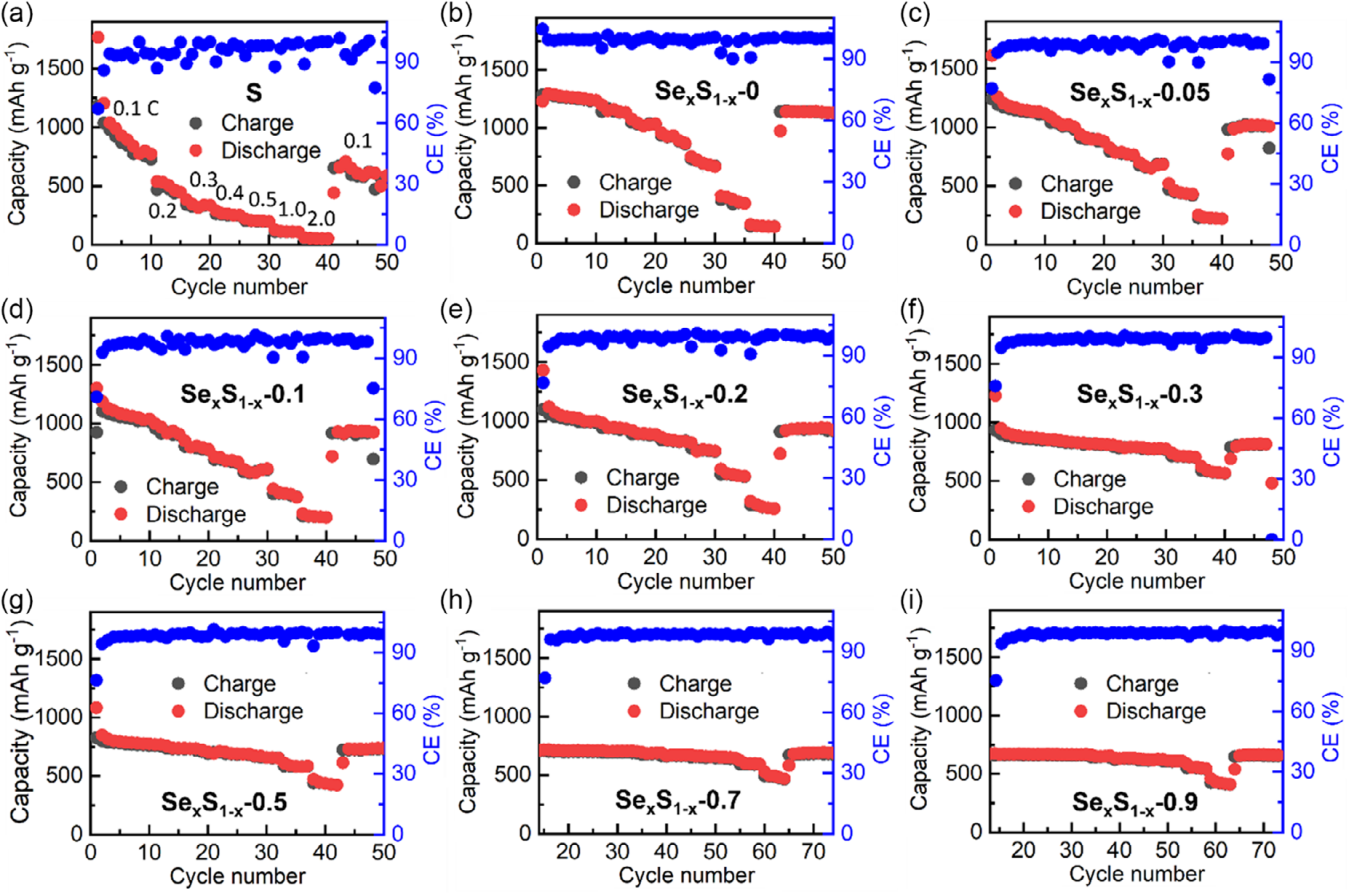
The summarized rate performances (0.1C, 0.2C, 0.3C, 0.4C, 0.5C, 1.0C, and 2.0 C) of the all‐solid‐state lithium batteries (ASSLBs) (Li–In/LYB/Se_*x*_S_1–*x*_) with different cathodes of: a) S and b–i) Se_*x*_S_1–*x*_ with *x* = 0 (b), *x* = 0.05 (c), *x* = 0.1 (d), *x* = 0.2 (e), *x* = 0.3 (f), g) *x* = 0.5, *x* = 0.7 (h), and *x* = 0.9 (i)) at room temperature (RT).

**Figure 4:**
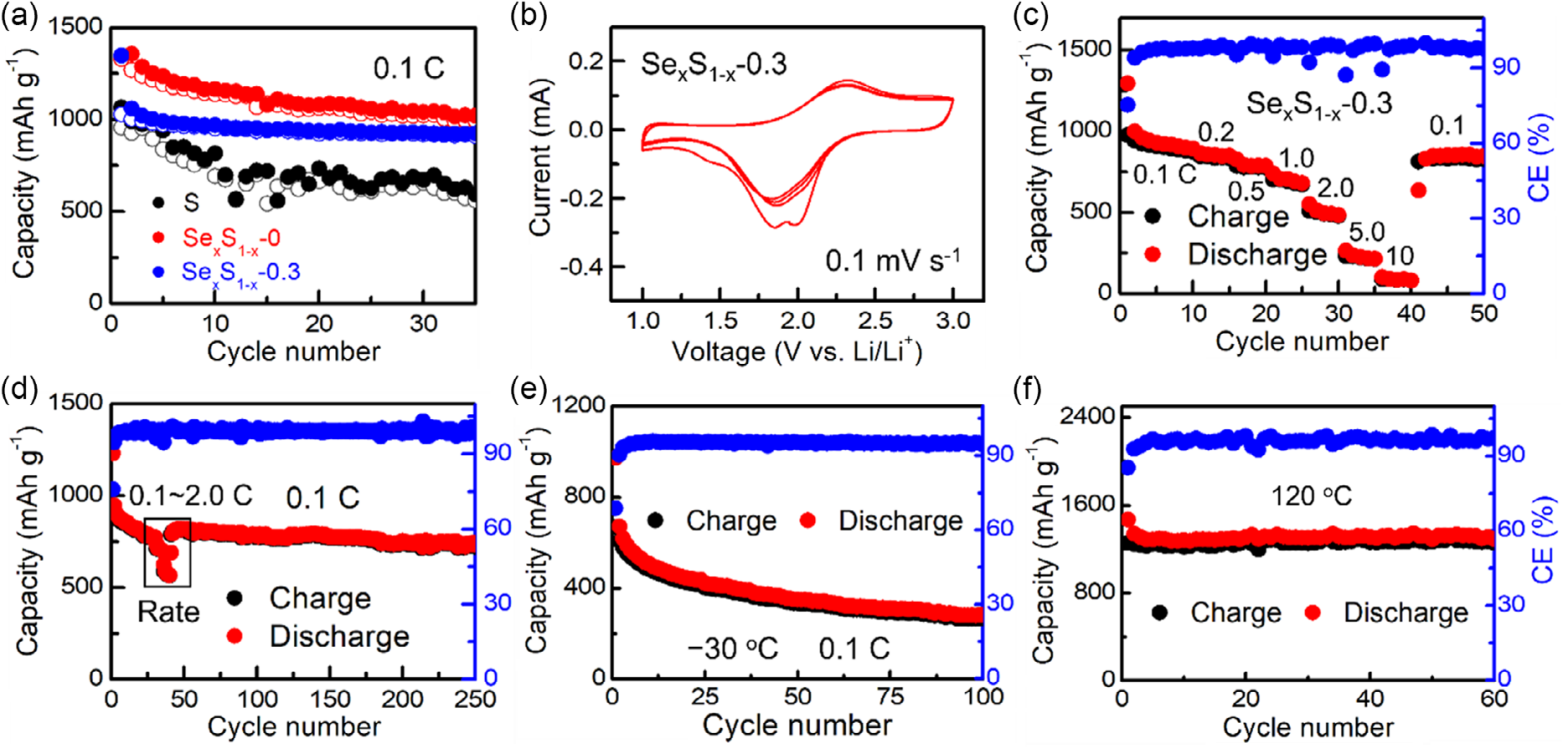
The performances of LYB‐based solid batteries (Li–In/LYB/Se_*x*_S_1–*x*_). a) Cycling stability of ASSLBs with S, Se_*x*_S_1–*x*_–0 and Se_*x*_S_1–*x*_‐0.3 cathodes at 0.1 C (RT). b) CV curves of ASSLB with Se_*x*_S_1–*x*_‐0.3 cathode. c) Rate performance (RT) of ASSLB with Se_*x*_S_1–*x*_‐0.3 cathode at different current densities (0.1–10C). d) Rate and cycling performance of ASSLB with Se_*x*_S_1–*x*_‐0.3 cathode at RT. e,f) Cycling performance of ASSLB with Se_*x*_S_1–*x*_‐0.3 cathode at −30 °C (e) and 120 °C (f).

To evaluate the battery performances of Se_*x*_S_1–*x*_ composite materials, LYB‐based ASSLBs were assembled by a simple cold‐pressing process, containing Li–In anode, LYB electrolyte, and S‐based cathode. The LYB‐based electrolyte layer (thickness: ≈270 μm) could realize compact morphology due to the good deformability of LYB sample (Figure S10, Supporting Information). The cathode composite sample was obtained by mixing Se_*x*_S_1–*x*_, LYB, and Ketjenblack (KB) through the ball‐milling method. Then, the discharge–charge and rate performances of commercial S and prepared Se_*x*_S_1–*x*_ (0 ≤ *x* ≤ 0.9) materials were tested at RT. From the summarized results (**Figure** **3**, Figure S11, Supporting Information), the prepared Se_*x*_S_1–*x*_ sample exhibited the better battery performances compared with commercial S cathode. In addition, along with the increase in the *x* value, the cycling and rate performances of Se_*x*_S_1–*x*_ cathodes were improved with the decreased capacities. It may be due to the decreased theoretical specific capacity and the improved electronic conductivity along with the addition of Se proportion in Se_*x*_S_1–*x*_. In view of the comprehensive discharge–charge and rate performances of cathode materials, the representative commercial S, Se_*x*_S_1–*x*_‐0, and Se_*x*_S_1–*x*_‐0.3 (Se_*x*_S_1–*x*_, *x* = 0.3) were selected for the further study. According to the cycling (**Figure** **4**a), cyclic voltammetry (CV) (Figure 4b, Figure S12, Supporting Information), discharge–charge (Figure S11a,b,f, Supporting Information), and rate (0.1C–2.0C) (Figure 3a,b,f) results of these assembled batteries, the trend of battery performance was S < Se_*x*_S_1–*x*_‐0 < Se_*x*_S_1–*x*_‐0.3, and the Se_*x*_S_1–*x*_‐0.3 cathode exhibited the smallest polarization, the most stable cycling performance, and the best rate performance. After that, the current density in the rate performance test of the Se_*x*_S_1–*x*_‐0.3 cathode was further improved (Figure 4c). When cycled at 0.1C, 0.2C, 0.5C, 1.0C, 2.0C, 5.0C, and 10C, the reversible capacities of the Se_*x*_S_1–*x*_‐0.3 cathode were 922, 859, 788, 698, 495, 226, and 84 mAh g^−1^, respectively. After the current density came back to 0.1C, the Se_*x*_S_1–*x*_‐0.3 cathode could maintain a capacity of 855 mAh g^−1^. Meanwhile, the Se_*x*_S_1–*x*_‐0.3 cathode exhibited stable cycling performance (0.1C) over 250 cycles after rate performance (0.1C–2.0C) test at RT (Figure 4d). After cycling process, the close contact of electrolyte/cathode layer in ASSLB with Se_*x*_S_1–*x*_‐0.3 cathode could be maintained for the excellent deformability of LYB; the cathode surface became uneven for the volume change of Se_*x*_S_1–*x*_‐0.3 (Figure S13–S14, Supporting Information); the Li–In anode can maintain the flat surface after cycling due to the uniform deposition of lithium; the Li–In/LYB layer can keep intimate contact due to the good deformability of LYB and Li–In anode (Figure S15, Supporting Information). In addition, the Se_*x*_S_1–*x*_‐0.3 cathode can realize good cycling performances and normal discharge–charge curves at −30 and 120 °C (Figure 4e,f, Figure S16, Supporting Information) due to the good electronic conductivity of Se_*x*_S_1–*x*_‐0.3 and the high thermal stability of LYB with good ionic conductivity. The ionic conductivities of LYB at −30 and 120 °C are consistent with the corresponding discharge–charge polarizations of ASSLB with Se_*x*_S_1–*x*_‐0.3 cathode (Figure S17, Supporting Information).

**Figure 5:**
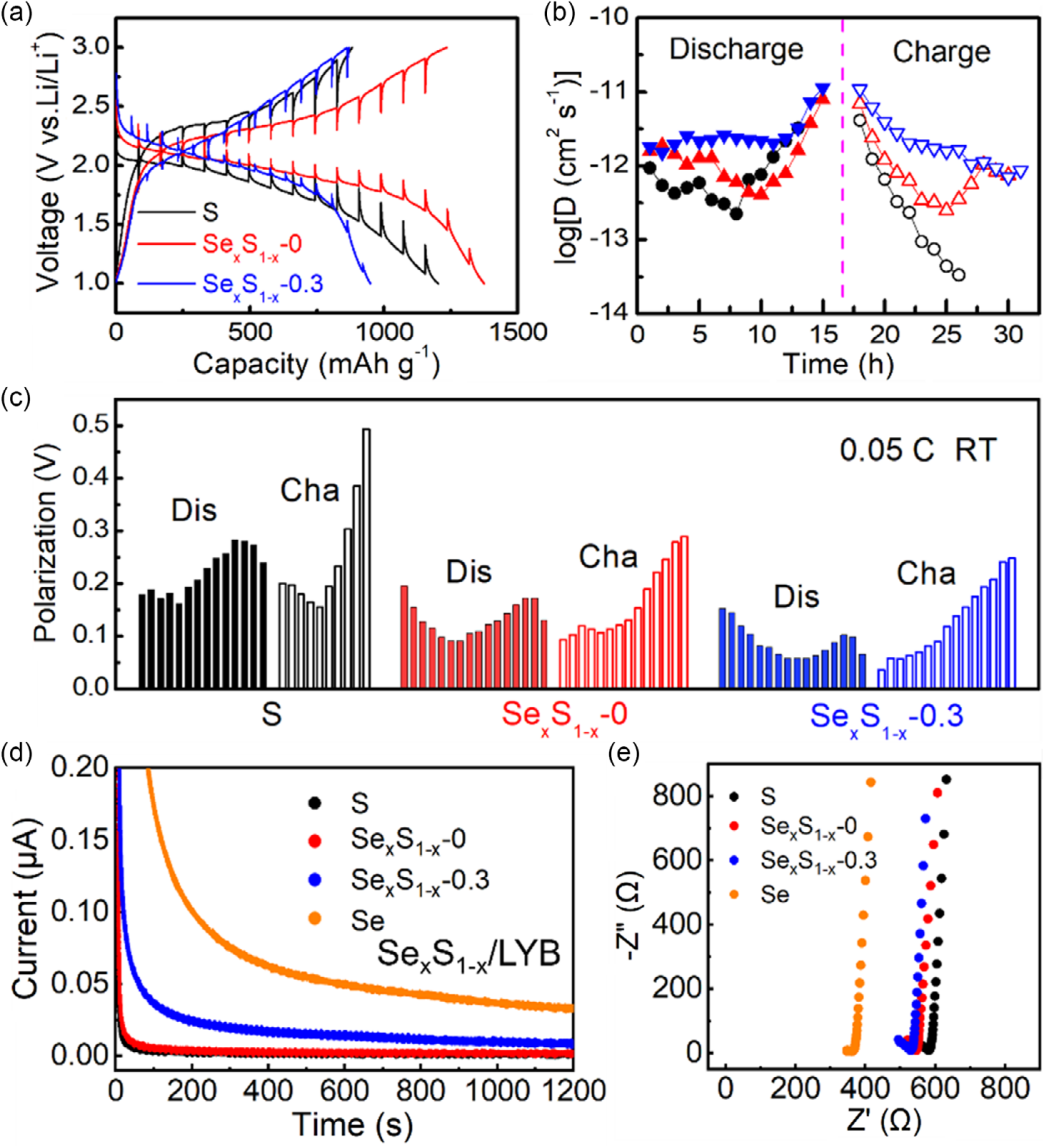
a) Galvanostatic intermittent titration technique (GITT) curves, b) *D*_Li_ values, and c) the summarized voltage polarizations of ASSLBs (Li–In/LYB/Se_*x*_S_1–*x*_) with S, Se_*x*_S_1–*x*_‐0 and Se_*x*_S_1–*x*_‐0.3 cathodes. d) Response currents at an applied bias (1.0 V) and e) electrochemical impedance spectroscopy results of LYB‐based composites mixed with S, Se_*x*_S_1–*x*_‐0, Se_*x*_S_1–*x*_‐0.3, and Se, respectively.

Galvanostatic intermittent titration technique (GITT) tests were conducted on the LYB‐based ASSLBs with different cathodes (S, Se_*x*_S_1–*x*_‐0, Se_*x*_S_1–*x*_‐0.3) at the same conditions (**Figure** **5**a). According to the calculated Li^+^‐diffusion coefficient (*D*_Li_) values (Figure 5b) and the aggregate voltage‐polarization values (Figure 5c) during discharge–charge processes, the trend of *D*_Li_ is S < Se_*x*_S_1–*x*_‐0 < Se_*x*_S_1–*x*_‐0.3, which is consistent with the trend of voltage polarization (Se_*x*_S_1–*x*_‐0.3 < Se_*x*_S_1–*x*_‐0 < S). Thus, the trend of electronic/ionic conductivity of these cathodes may be S < Se_*x*_S_1–*x*_‐0 < Se_*x*_S_1–*x*_‐0.3. In addition, EIS tests were conducted on these cells, and the discharge–charge impedances of the ASSLBs with S, Se_*x*_S_1–*x*_‐0, and Se_*x*_S_1–*x*_‐0.3 cathodes decreased successively (Figure S18, Supporting Information), corresponding to the GITT results. From the EIS spectra, the interfacial impedance is not obvious, indicating that LYB possesses good compatibility with the S‐based cathode and that the electrolyte/cathode layer possesses close contact for the good deformability of LYB.

To evaluate the electronic/ionic conductivity of these S‐based cathode materials, S, Se_*x*_S_1–*x*_‐0, Se_*x*_S_1–*x*_‐0.3, and Se were mixed with LYB by ball‐milling method, and these composites were characterized by chronoamperometry and EIS tests. According to the obtained direct current polarization curves and Nyquist plots of these composites (Figure 5d–e), the Se/LYB sample exhibits the best electronic/ionic conductivity, indicating that the introduction of Se is beneficial for improving the electronic/ionic conductivities of Se_*x*_S_1–*x*_ materials. The Se_*x*_S_1–*x*_‐0/LYB sample possesses higher ionic conductivity than the S/LYB sample, and they have similar electronic conductivities; the Se_*x*_S_1–*x*_‐0.3/LYB sample exhibits higher electronic conductivity than the S/LYB and Se_*x*_S_1–*x*_‐0/LYB samples, and the ionic conductivity of the Se_*x*_S_1–*x*_‐0.3/LYB sample is close to that of the Se_*x*_S_1–*x*_‐0/LYB sample. Thus, for the Se_*x*_S_1–*x*_ materials, the improvement in battery performance mainly comes from the higher electronic/ionic conductivity of Se_*x*_S_1–*x*_ compared with the S sample.

**Figure 6:**
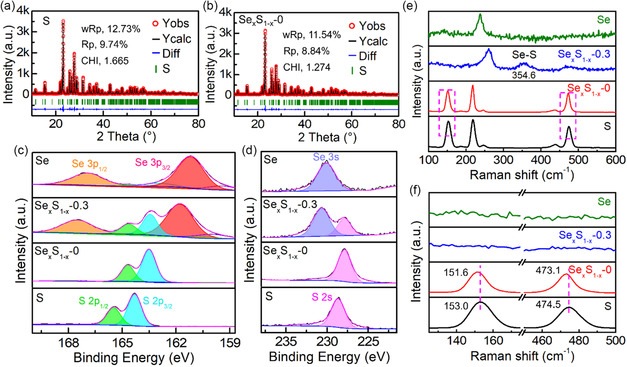
a,b) Rietveld refinements of XRD patterns of S (a) and Se_*x*_S_1–*x*_‐0 (b). c,d) The XPS spectra and e,f) Raman spectra of S, Se_*x*_S_1–*x*_‐0, Se_*x*_S_1–*x*_‐0.3, and Se.

To study the mechanism for the enhancement of ionic conductivity of the Se_*x*_S_1–*x*_‐0 sample, the structures of S and Se_*x*_S_1–*x*_‐0 were further analyzed by XRD Rietveld refinement according to the corresponding XRD results (**Figure** **6**a,b). The obtained cell parameters of Se_*x*_S_1–*x*_‐0 are larger than those of the S sample (Table S1, Supporting Information), thus, the Se_*x*_S_1–*x*_‐0 sample may have more vacancy defects from the melting process, which is beneficial to electronic/ionic transport and can explain the color change from S (yellow) to Se_*x*_S_1–*x*_‐0 (black). The S‐based samples (S, Se_*x*_S_1–*x*_‐0, Se_*x*_S_1–*x*_‐0.3, and Se) were further studied by XPS and Raman. According to the XPS results, the Se_*x*_S_1–*x*_‐0.3 can be confirmed as the composite of Se and S with zero valence, and the shifts to the lower binding energy of XPS peaks (S 2*s* and S 2*p*) of S sample after melting process (Se_*x*_S_1–*x*_‐0) may be attributed to the addition of defects (Figure 6c,d).^[^ ^50^ ^]^ From the Raman results, the new Raman peak (354.6 cm^−1^) of Se_*x*_S_1–*x*_‐0.3 sample may be assigned to the stretching vibration of Se–S bond (Figure 6e).^[^ ^51^ ^]^ In addition, the S and Se_*x*_S_1–*x*_‐0 samples possess the similar Raman spectra, and some Raman peaks of Se_*x*_S_1–*x*_‐0 exhibit slight deviation to the left, which can demonstrate the more abundant defects of Se_*x*_S_1–*x*_‐0 sample (Figure 6f).^[^ ^52^ ^]^ In short, the Se_*x*_S_1–*x*_ materials possess better battery performances compared with the S sample, which may be due to the improvement in electronic/ionic conductivity after the introduction of Se and the increase in vacancy defects after the melting process.

## 3 Conclusion

High performance of LYB‐based solid batteries can be realized by the design of S‐based cathode. To improve the electronic/ionic conductivity of S, a series of Se_*x*_S_1–*x*_ (0 ≤ *x* ≤ 0.9) composites were prepared, and the sample Se_*x*_S_1–*x*_‐0.3 was selected for the comprehensive advantages of good cycling performance (250 cycles), high capacity, and excellent rate performance (0.1–10 C) at RT. The high performance of Se_*x*_S_1–*x*_‐0.3 cathode can be attributed to the improved electronic/ionic conductivity and abundant defects from the melting process (380 °C). In addition, the Se_*x*_S_1–*x*_‐0.3 battery based on LYB can be operated at a low temperature of −30 °C and a high temperature of 120 °C. Therefore, the improvements in LYB‐based solid batteries with Se–S composite cathodes are significant for the development of ASSLBs.

## 4 Experimental Section

4.1

4.1.1

##### Chemicals

Li_2_CO_3_ (99.99%) and P_2_S_5_ (99%) were purchased from Shanghai Meryer Chem. Technol. Co. Ltd. Y_2_O_3_ (99.999%) was purchased from Beijing HWRK Chem. Co. Ltd. Se powder (99.9%), NH_4_Br (99.0%), and HBr (48 wt% in water) were purchased from Shanghai Aladdin Biochem. Technol. Co. Ltd. Li_2_S (99.9%) was purchased from Beijing Innochem Science & Technology Co. Ltd. Lithium (Li) metal was purchased from China Energy Lithium Co. Ltd. Indium (In) metal was purchased from Changsha HAOJIA New Material Co. Ltd. KB (Carbon ECP200L) was purchased from Lion Corporation. S powder (99.5%) was purchased from Alfa Aesar (China) Chemicals Co. Ltd.

##### Synthesis of SE

LYB was prepared based on our previous work with a vacuum‐evaporation‐assisted method.^[^ ^45^ ^]^ 6 mmol Li_2_CO_3_ and 2 mmol Y_2_O_3_ were added in a beaker, and then poured appropriate HBr solution slowly into this beaker with heating and stirring. When all the solids were disappeared in solution, excessive NH_4_Br and enough deionized water were added to prepare a homogeneous solution. It was kept heating and stirring until the solid precursor was obtained. Then, the solid sample was heated (400 °C for 100 min, then 450 °C for 120 min) under vacuum and argon gas (Ar) protection to obtain LYB. Then, the prepared LYB sample was transferred into an argon glove box for storage and use.

Referring to the reported work, LPS was obtained by ball‐milling and annealing processes.^[^ ^53^ ^]^ Li_2_S and P_2_S_5_ with a molar ratio of 7/3 were weighed in an argon glove box and then treated by ball‐milling (480 rpm, 20 h) under the protection of Ar. After each 15 min working state, the ball‐milling was interrupted to avoid overheating. Then, the obtained mixed powder was cold‐pressed and annealed (250 °C, 3 h) under an Ar atmosphere. After cooling, the LPS sample could be obtained.

##### Synthesis of Se_x_S_1–x_ Composites

The stoichiometric ratio of Se and S powders were mixed by grinding for 10 min and then transferred into a glass bottle. This bottle was sealed and calcined at 380 °C for 10 h. The Se_*x*_S_1–*x*_ (0 ≤ *x* ≤ 0.9) sample was obtained and ground for the next use.

##### Materials Characterization

XRD was conducted by a Rigaku MiniFlex600 diffractometer (Cu Kα) and Rigaku Smart Lab 3 kW (Cu Kα) diffractometer. The obtained spectra were analyzed by Rietveld refinement using GSAS‐EXPGUI.^[^ ^54^ ^]^ The SEM images were collected by an instrument (JEOL JSM‐7800F) attached to the energy disperse spectrometer. The samples were characterized by XPS (Thermo Scientific, ESCALAB 250Xi (Al Kα)) and Raman spectroscopy (SR‐500I‐A, TEO, USA, 532 nm laser).

##### Electrochemical Characterizations

The In/LYB/In battery was prepared by a simple cold forming method. First, the 120 mg of LYB powder was pressed (4 ton) in a mold (pellet, diameter: 10 mm; thickness: ≈0.5 mm). Then, two tailored In foils were pressed on the two sides of LYB pellet as blocking electrodes. The assembled In/LYB/In cell was tested by EIS and the ionic conductivity was calculated. All measurements were performed on an electrochemical workstation (Autolab PGSTAT302N). For the Li/LYB/Li and Li–In/LYB/Li–In cells, the discharge–charge and cycling performances were tested (0.1 mA cm^−2^).

In this work, all‐solid‐state Li–In/LYB/Se_*x*_S_1–*x*_ cells were prepared by cold‐pressing method. To realize effective ionic/electronic conduction of the cathode composite, Se_*x*_S_1–*x*_, LYB (ionic conductor), and KB (conductive agent) were mixed by ball‐milling method (480 rpm for 4 h) under the protection of Ar. Then, cathode composite powder, LYB powder, and Li–In alloy were cold‐pressed (4 ton) in a mold (diameter: 10 mm). For the electrochemical tests, the obtained Li–In/LYB/Se_*x*_S_1–*x*_ pellets were encapsulated in CR2032 coin cells. The CV curves of Li–In/LYB/Se_*x*_S_1–*x*_ cells were collected on a CHI660E instrument. In addition, these assembled Li–In/LYB/Se_*x*_S_1–*x*_ cells were discharged and charged on an instrument (LANHE CT2001A). For the test of rate performance, the Li–In/LYB/Se_*x*_S_1–*x*_ cells were cycled at different current densities (Theoretical capacity, S, Se_*x*_S_1–*x*_–0: 1675 mAh g^−1^; Se_*x*_S_1–*x*_‐0.05: 1560 mAh g^−1^; Se_*x*_S_1–*x*_‐0.1: 1460 mAh g^−1^; Se_*x*_S_1–*x*_‐0.2: 1293 mAh g^−1^; Se_*x*_S_1–*x*_‐0.3: 1161 mAh g^−1^; Se_*x*_S_1–*x*_‐0.5: 963 mAh g^−1^; Se_*x*_S_1–*x*_‐0.7: 823 mAh g^−1^; and Se_*x*_S_1–*x*_‐0.9: 718 mAh g^−1^). GITT tests of Li–In/LYB/Se_*x*_S_1–*x*_ cells were conducted at the same conditions (0.05 C for 1 h; standing for 4 h). During discharge–charge processes, EIS tests were conducted on Li–In/LYB/Se_*x*_S_1–*x*_ cells (0.01 Hz to 1 MHz).

## Conflict of Interest

The authors declare no conflict of interest.

## Supporting information

Supplementary Material

## Data Availability

The data that support the findings of this study are available from the corresponding author upon reasonable request.
