# Supplementary material for: High‐Performance Se–S Composite Cathode Rich in Defects for Wide‐Temperature Solid‐State Lithium Batteries
Source: Small Sci. 2023 Nov 20;3(12):2300134. doi: 10.1002/smsc.202300134 (PMC11935919; doi:10.1002/smsc.202300134)
Supplement: Supplementary file 1 — Supplementary Material [file SMSC-3-2300134-s001.pdf]

Supporting Information

High-Performance Se–S Composite Cathode Rich in Defects for Wide-Temperature Solid-State Lithium Batteries

Xiaomeng Shi, Zhichao Zeng, Yongqing Wen, Hongtu Zhang, Yabin Zhang, and Yaping Du**

* Corresponding author

E-mail address: ypdu@nankai.edu.cn (Y. Du); zeng@mail.nankai.edu.cn (Z. Zeng)

Table of contents

Figure S1. The EDS result of LYB.

Figure S2-S4. The electrochemical performances of LYB.

Figure S5-S6. The photos of S and Se_xS_{1-x} composites.

Figure S7-S8. The XRD patterns of S and Se_xS_{1-x} composites.

Figure S9. The SEM and EDS results of Se-S composites.

Figure S10. The SEM results of LYB-based ASSLB.

Figure S11-S12. The battery performances of S and Se_xS_{1-x} cathodes.

Figure S13-S15. SEM results of ASSLBs with Se_xS_{1-x}-0.3 cathode.

Figure S16. The battery performances of Se_xS_{1-x}-0.3 cathode at −30 °C and 120 °C.

Figure S17. The EIS spectra of LYB at −30 °C and 120 °C.

Figure S18. The EIS spectra of ASSLBs with S and Se_xS_{1-x} cathodes.

Table S1. The cell parameters of S and Se_xS_{1-x}-0.

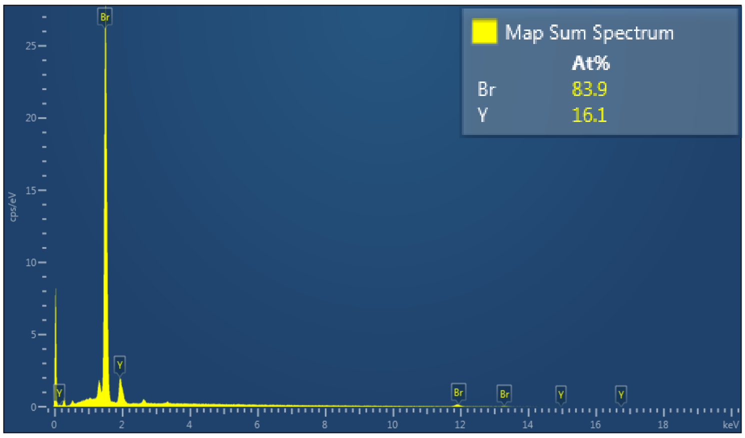

Figure S1. The EDS result of LYB.

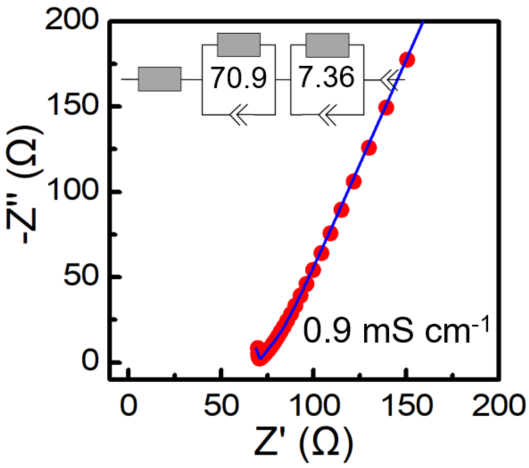

Figure S2. The Nyquist plot of assembled In/LYB/In cell.

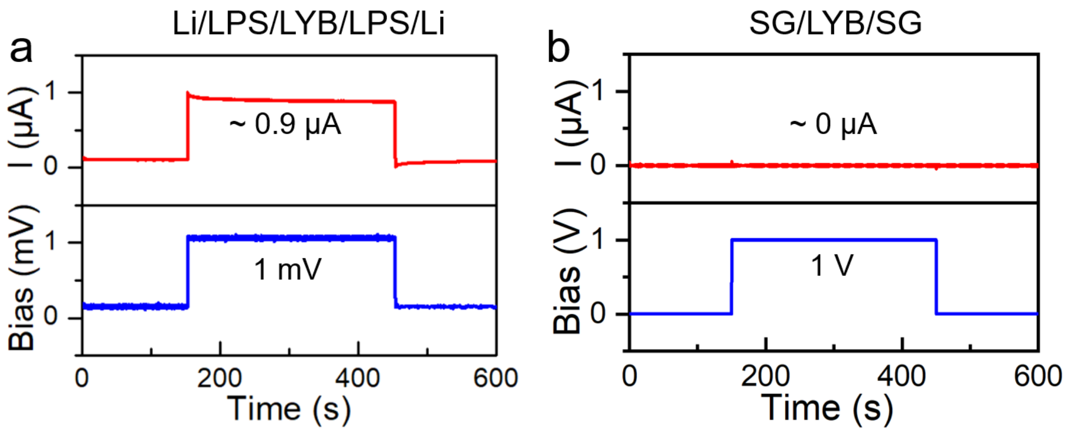

Figure S3. The transient current behaviors on (a) Li/LPS/LYB/LPS/Li cell (bias, 1 mV) and (b) SG/LYB/SG cell (bias, 1 V).

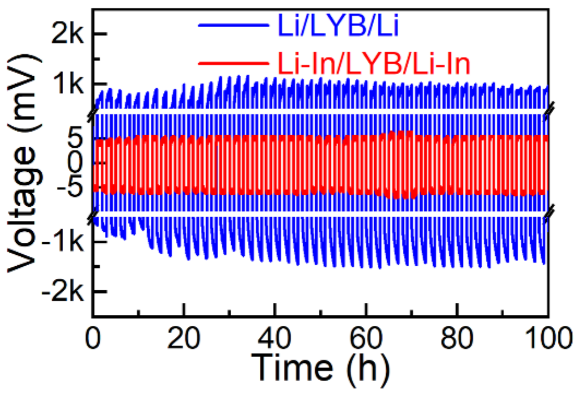

Figure S4. Electrochemical performance of the symmetric Li/LYB/Li and Li-In/LYB/Li-In cells at 0.1 mA cm^{-2}.

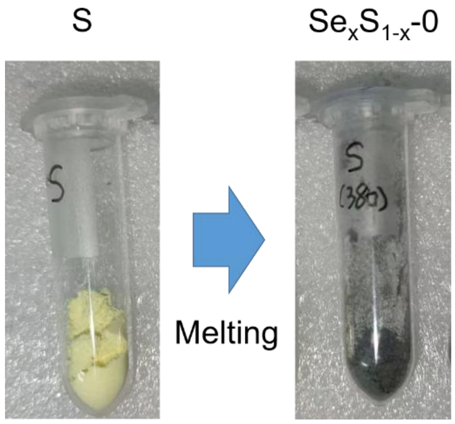

Figure S5. The photos of commercial S and the S sample after melting process (Se$_x$S$_{1-x}$-0).

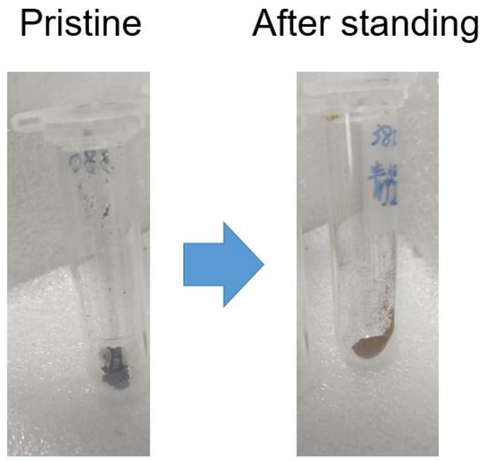

Figure S6. The photos of Se$_x$S$_{1-x}$-0.3 sample before and after standing process.

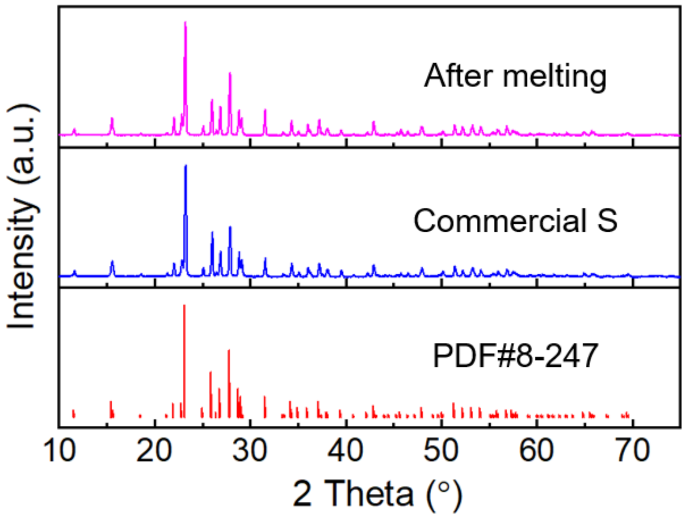

Figure S7. The XRD patterns of commercial S and the S sample after melting process (Se_xS_{1-x}-0).

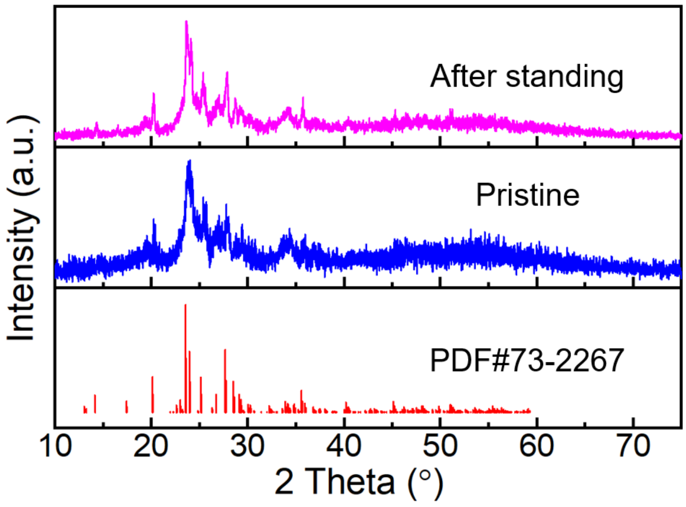

Figure S8. The XRD patterns of Se_xS_{1-x}-0.3 sample before and after standing process.

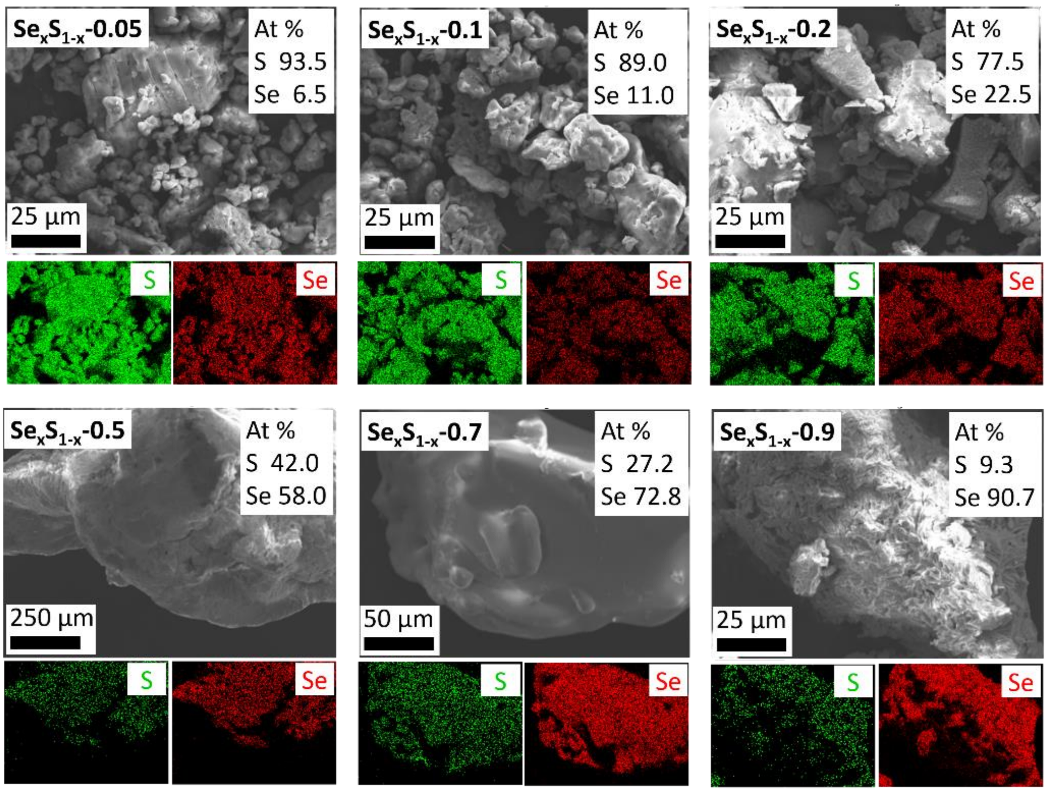

Figure S9. The summarized SEM/EDS results of Se_xS_{1-x} ($0 < x \leq 0.9$) materials.

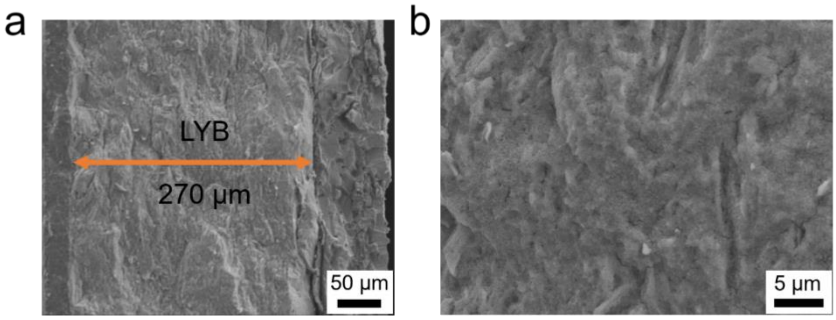

Figure S10. The cross-section SEM images of (a) the all-solid-state lithium batteries (ASSLBs) (Li-In/LYB/Se_xS_{1-x}) and (b) the enlarged LYB electrolyte layer.

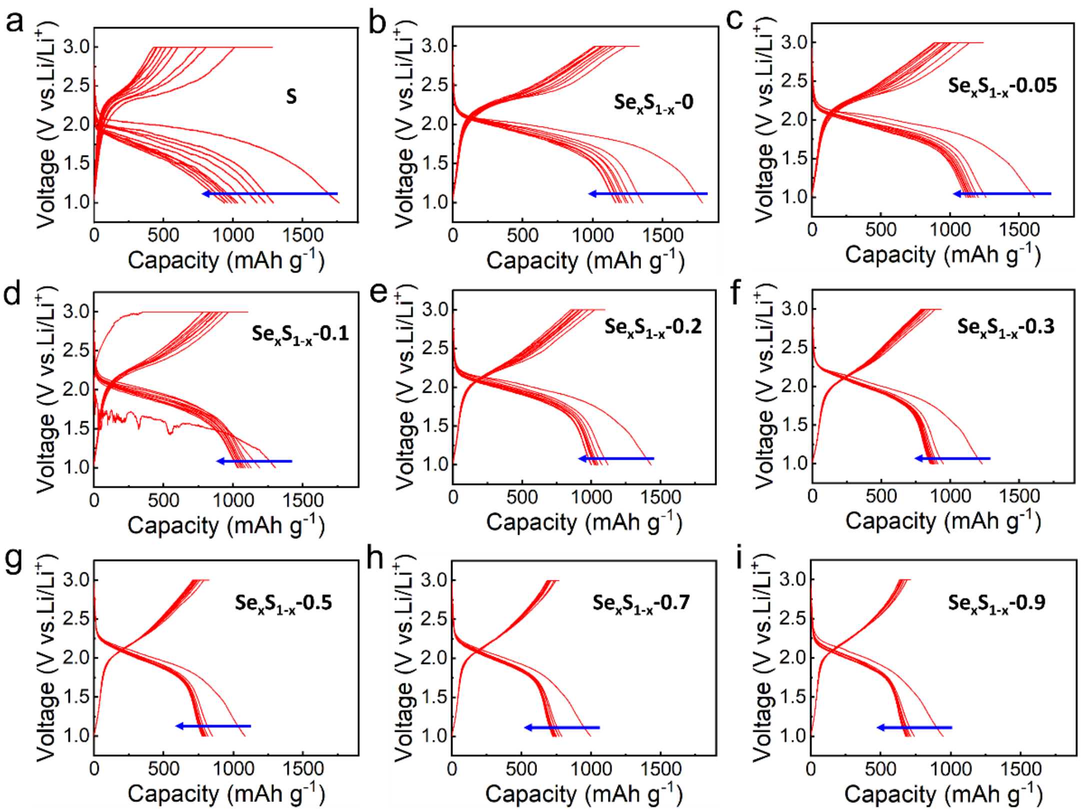

Figure S11. The summarized discharge-charge curves of the ASSLBs (Li-In/LYB/Se_xS_{1-x}) with cathodes of (a) S and (b-i) Se_xS_{1-x} ($0 \leq x \leq 0.9$) at RT.

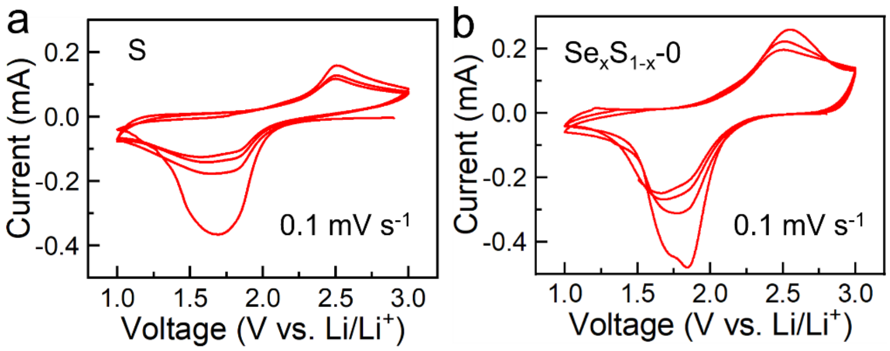

Figure S12. The CV curves of the ASSLBs (Li-In/LYB/Se_xS_{1-x}) with (a) S and (b) Se_xS_{1-x}-0 cathodes.

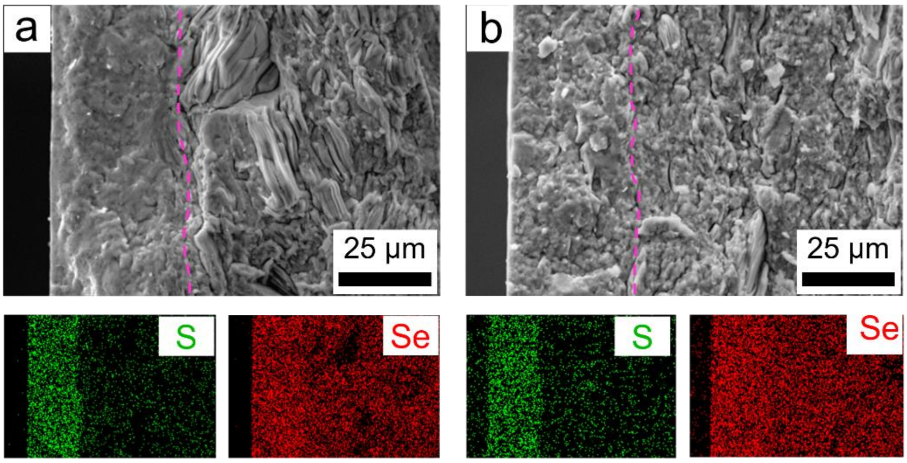

Figure S13. The cross-section SEM images of (a) pristine and (b) cycled electrolyte/cathode layers of ASSLBs (Li-In/LYB/Se_xS_{1-x}) with Se_xS_{1-x}-0.3 cathode and the corresponding EDS mapping of S and Se.

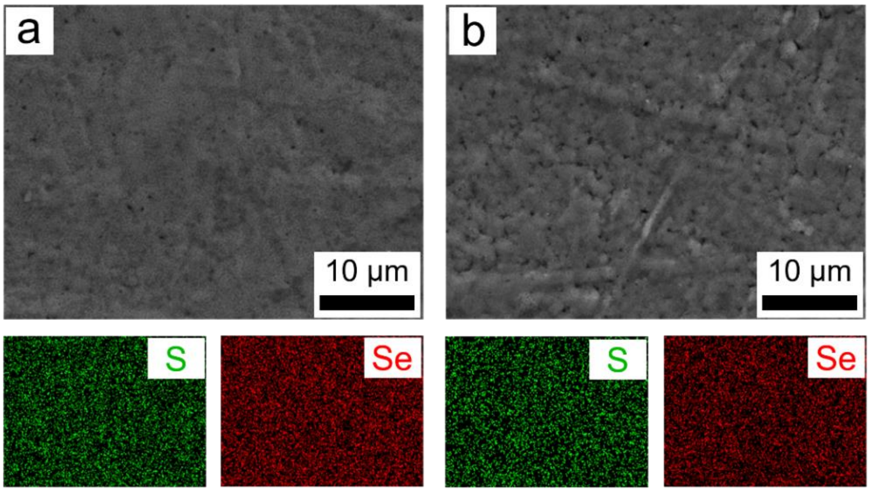

Figure S14. The top-view SEM images of (a) pristine and (b) cycled cathode layers of ASSLBs (Li-In/LYB/Se_xS_{1-x}) with Se_xS_{1-x}-0.3 cathode and the corresponding EDS mapping of S and Se.

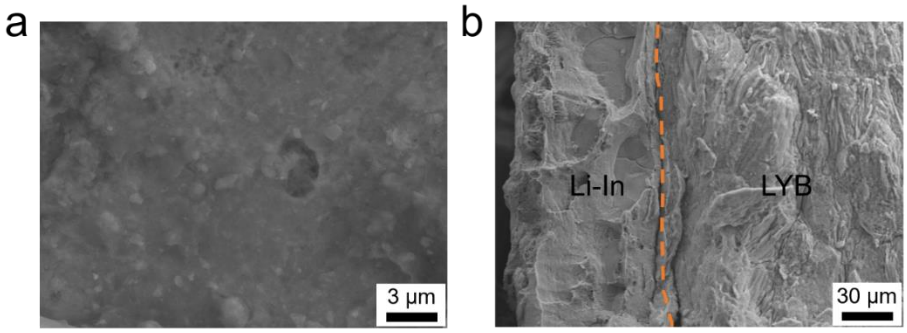

Figure S15. (a) The SEM image of cycled Li-In anode. (b) The cross-section SEM image of cycled Li-In/LYB layer in ASSLB (Li-In/LYB/Se_xS_{1-x}) with Se_xS_{1-x}-0.3 cathode.

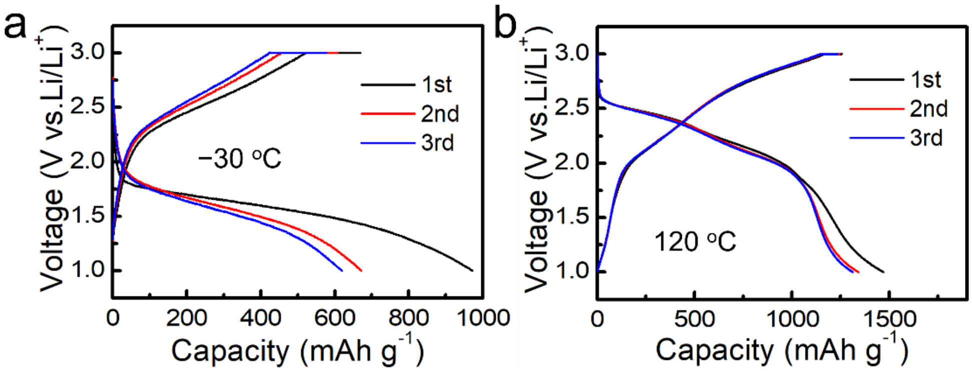

Figure S16. The discharge-charge curves of ASSLBs (Li-In/LYB/Se_xS_{1-x}) with Se_xS_{1-x}-0.3 cathode at (a) −30 °C and (b) 120 °C.

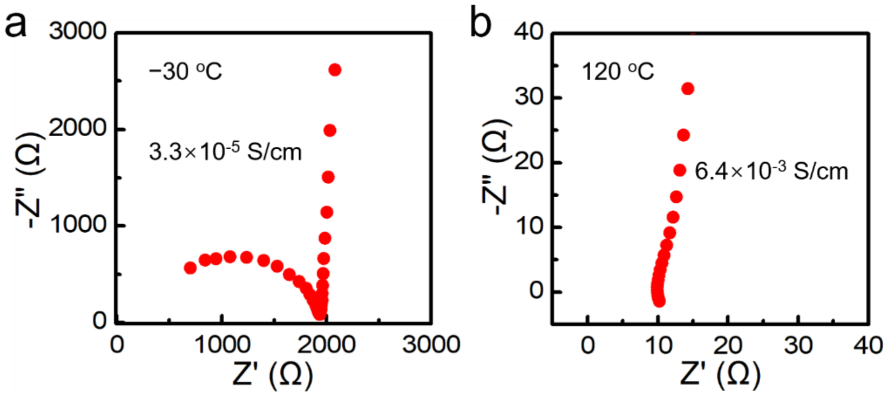

Figure S17. The EIS spectra of LYB based symmetrical cell measured at (a) −30 °C and (b) 120 °C.

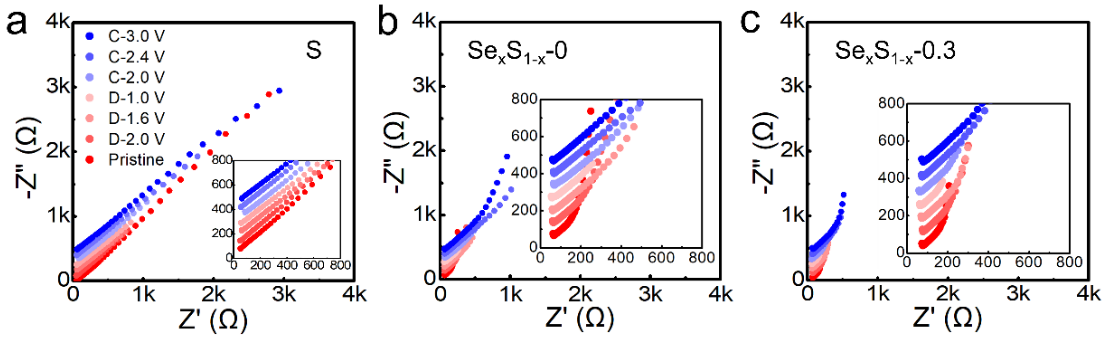

Figure S18. EIS spectra of ASSLBs (Li-In/LYB/Se_xS_{1-x}) with (a) S, (b) Se_xS_{1-x}-0 and (c) Se_xS_{1-x}-0.3 cathodes during discharging and charging processes.

Table S1 The cell parameters of S and Se_xS_{1-x}-0 obtained from XRD Rietveld refinement results.

Parameters	S	Se_xS_{1-x}-0
a	10.4965	10.5014
b	12.9095	12.9138
c	24.5480	24.5577
Volume	3326.40	3330.34
